# What Happens under Mr. Holland's System

**Published:** 1903-03-07

**Authors:** 


### WHAT HAPPENS UNDER MR. HOLLAND'S SYSTEM.

### From a Nurse's Point of View.

A night sister, with many years' experience, has sent us the following letter: May I venture, as a nurse, to give two examples, typical, however, of many of the disturbances caused in a ward by the admission of these patients. At a large hospital I was night nurse in sole charge of a ward of 28 men, medical cases, many seriously, some dangerously ill. Two amongst them were cases of advanced mitral disease, one a pneumonia, nearing his crisis, who had just closed his eyes for the first time for 48 restless hours. An "ambulance," pushed by a porter who was nearly bent double over a gesticulating shouting "admission" appeared at the door, then stopped while the porter tried to collect the waving arms and legs, and drag his charge on to the ambulance from which he had nearly slipped in his struggles.

He called to me to help him, and said he had had twice to fling himself on the top of the patient and hold him down while coming up in the lift. The patient was so violent he had to be tied down in bed, and it was almost impossible to keep even a square inch of blanket on him during the process of being undressed and put to bed. It is simply heartbreaking, after all one's care, to "gradually wake" one's heart cases and to spare them every excitement or exertion, after begging the men well enough to leave their beds to speak *sotto voce* and creep noiselessly about the ward for fear of waking the pneumonia from the much longed for—yes, prayed for, sleep—to have a drunken epileptic, as the noisy admission proved to be, turned into a ward where rest and quiet are often the essential part of the treatment. Another true picture: A women's surgical ward under my care as night sister. Among the women a highly nervous "ovarian"—a day or two operated upon. A filthy creature, roaring, literally roaring drunk, was admitted. She had a large scalp wound, and her matted hair, soaked with blood, was hanging loose over her clothes. This lady heralded her approach by shouting, "I'm bleedin' ter death, I tell ye! I'll have the lawr o' them as did it, . . ." etc. She was brought in by the police, and was a well-known drunken brawler. She constantly got out of bed and frightened the women terribly. Surely if the powers that be realised how utterly unfit these cases are for admission to any but single-bedded (or emergency) wards, they would at once try to provide the necessary accommodation. Even the doctors do not grasp the situation; they pay their visit or visits and go. The nurse stays, and for hours tries to soothe her disturbed and frightened charges, tries to keep the noisy new admission under some control and some semblance of decency, and dashes off between whiles to take temperatures, give medicines, refill hot bottles, and apply fomenta tions, to say nothing of giving drinks and "screens" to her patients.

There are some noisy cases one must put up with, but in the kind of case under discussion the noise and violence would probably pass away in 24 hours, and then admission to a ward would be necessary and justifiable, but to admit them in the condition I have described is cruel to the other patients and lessens their chances of recovery.

